# Trends in Gender Disparities in Surgical Experience: A National Clinical Database Study

**DOI:** 10.1002/ags3.70080

**Published:** 2025-08-23

**Authors:** Chie Tanaka, Hiroyuki Yamamoto, Sachiyo Nomura, Emiko Kono, Hideki Ueno, Yoshihiro Kakeji, Ken Shirabe

**Affiliations:** ^1^ Department of Surgery (Gastrointestinal Surgery) Nagoya University Hospital Nagoya Japan; ^2^ Department of Healthcare Quality Assessment, Graduate School of Medicine The University of Tokyo Tokyo Japan; ^3^ Department of Clinical Pharmaceutical Sciences Hoshi University Tokyo Japan; ^4^ Department of Gastrointestinal Surgery, Graduate School of Medicine The University of Tokyo Hospital Tokyo Japan; ^5^ Department of General and Gastroenterological Surgery Osaka Medical and Pharmaceutical University Takatsuki Japan; ^6^ Department of Surgery National Defense Medical College Tokorozawa Japan; ^7^ Division of Gastrointestinal Surgery, Department of Surgery Kobe University Graduate School of Medicine Kobe Japan; ^8^ Department of General Surgical Science Graduate School of Medicine, Gunma University Maebashi Japan

**Keywords:** gender disparity, National Clinical Database, surgical experience

## Abstract

**Background:**

We previously reported the gender disparities in surgical procedures between male and female surgeons using National Clinical Database covering more than 95% of all operations performed in Japan. This study aims to examine the changes in gender disparity in the surgical experience of gastrointestinal surgeons in Japan before and after implementation of measures by the Japanese Society of Gastrointestinal Surgery to address gender inequality commenced in 2021.

**Methods:**

We conducted a nationwide retrospective study to compare the number of operations performed by male and female surgeons using National Clinical Database. The number of operations per surgeon was calculated based on every 2 years of a surgeon's experience, and a comparison was made between male and female surgeons. The years selected for analysis were 2015, 2019, and 2023.

**Results:**

Almost no gender differences were observed in the number of low‐difficulty surgeries. For medium‐difficulty surgeries, the number performed by female surgeons showed an improving trend over the study period, yet some disparities remained. The number of high‐difficulty surgeries (low anterior resection and pancreaticoduodenectomy) performed by male surgeons was higher than the number performed by female surgeons, except for protrusions. This disparity remained unchanged over the study period.

**Conclusions:**

The efforts of the Japanese Society of Gastrointestinal Surgery has been shown to be effective in mitigating gender disparities in the number of surgeries performed. High‐difficulty surgeries have emerged as the primary target for further improvement initiatives.

## Abstract 2

This nationwide study evaluated gender disparities in gastrointestinal surgical experience in Japan before and after 2021, when the Japanese Society of Gastrointestinal Surgery implemented measures to address gender inequality. While female surgeons showed increased involvement in medium‐difficulty procedures, disparities in high‐difficulty surgeries persisted, highlighting the need for continued efforts targeting equitable surgical opportunities.

## 1 Introduction

In the field of surgery, there are few female surgeons in leadership positions. One reason for this involves gender disparities in surgical training [1, 2]. Addressing gender disparity in surgery is a moral obligation and will likely lead to improvements in patient care, financial performance, innovation, and risk assessment [3, 4]. Both female and male surgeons are expected to acquire a similar level of surgical skill and have leading roles in surgical practice. Because surgical practice has an important impact on a surgeon's career, identifying and eliminating differences in the surgical experience between male and female surgeons has important implications for female surgeons holding leadership positions.

We investigated the disparity in surgical procedures between male and female surgeons using the National Clinical Database (NCD) 2013–2017 [5]. According to this report, female surgeons have less surgeries performed than male surgeons in Japan, and this gap tends to widen with more years of experience, especially for medium‐ and high‐difficulty operations. Accordingly, the Japanese Society of Gastrointestinal Surgery (JSGS) established a committee for gender equality and a female quota for council members and has made efforts to improve gender equality from 2021. In 2023, the JSGS issued the “Hakodate Declaration”, committing to gender equality in surgery and reviewing operative case numbers by gender after hosting a special session on gender bias [6].

The purpose of this study was to examine the change in gender disparity regarding surgeries performed among gastrointestinal surgeons in Japan, using the NCD, before and after initiatives of the JSGS.

## 2 Methods

### 2.1 Study Design and Ethics

We conducted a nationwide retrospective study using the NCD to compare the number of operations performed by male and female surgeons. The NCD is a nationwide surgical registration system in Japan that is linked to the JSGS board certification system and contains information on more than 95% of all operations performed in Japan. This study was approved by the ethics committee of Nagoya University Graduate School of Medicine (approval number: 2024‐0036). All procedures were in accordance with the ethical standards of the responsible committee on human experimentation and were in compliance with the Helsinki Declaration of 1964 and later versions.

### 2.2 Selection of Surgical Procedures and Assessment of Number of Surgical Procedures

Among the operations performed by JSGS members, the following elective surgeries were selected for inclusion in this cross‐sectional study: appendectomy and cholecystectomy, defined as low‐difficulty surgeries by the JSGS training curriculum for gastroenterological surgeons; right hemicolectomy and distal gastrectomy, defined as medium‐difficulty surgeries; and low anterior resection and pancreaticoduodenectomy, defined as high‐difficulty surgeries. The years of analyses were selected at 4‐year intervals: 2015, 2019, and 2023.

Surgeons' years of experience were calculated as the number of years from the date of medical registration. Gender information was obtained by matching the medical registration number with JSGS member records containing gender information. The number of years from the date of registration was divided into 2‐year increments for all years after medical registration. Surgeons who had not performed as the operator or assistant for over 3 months were excluded from the analyses.

### 2.3 Study Endpoints

The primary outcome was the number of operations per surgeon, categorized by gender and years of experience. The secondary outcome was the number and percentage of operations performed by male and female surgeons, categorized by gender and years of experience.

### 2.4 Statistical Analyses

The primary outcome was the number of operations per surgeon, categorized by gender and years of experience. To exclude surgeons who had not performed as the operator or assistant for over 3 months, the year was divided into four quarters. The number of operations for the year was then calculated as follows:

$$X=X\;\left(\text{quarter}\;1\right)+X\;\left(\text{quarter}\;2\right)+X\;\left(\text{quarter}\;3\right)+X\;\left(\text{quarter}\;4\right)$$

The number of surgeons for the year was calculated as follows:

$$Y=\left(Y\;\left(\text{quarter}\;1\right)+Y\;\left(\text{quarter}\;2\right)+Y\;\left(\text{quarter}\;3\right)+Y\;\left(\text{quarter}\;4\right)\right)/4$$

The number of operations per surgeon per year was calculated as follows:

$$\begin{array}{c} Z=X\;\left(\text{quarter}\;1\right)/Y\;\left(\text{quarter}\;1\right)+X\;\left(\text{quarter}\;2\right)/Y\;\left(\text{quarter}\;2\right)\\ +X\;\left(\text{quarter}\;3\right)/Y\;\left(\text{quarter}\;3\right)+X\;\left(\text{quarter}\;4\right)/Y\;\left(\text{quarter}\;4\right) \end{array}$$

The number of operations per surgeon for each 2‐year period of a surgeon's experience was calculated by summing the annual number of operations per surgeon.

The chi‐square test was used to compare the values of categorical variables. *p* < 0.05 was considered to indicate a significant difference. Data handling and statistical analyses were performed using Stata 18 (StataCorp LLC, College Station, TX, USA), JMP software, version 17 (SAS Institute Inc., Cary, NC, USA), and Microsoft Excel (Office Professional Plus 2021; Microsoft Corporation, Redmond, WA, USA).

## 3 Results

### 3.1 Number and Percentage of Female Surgeons in the JSGS by Years of Experience

**FIGURE 1:**
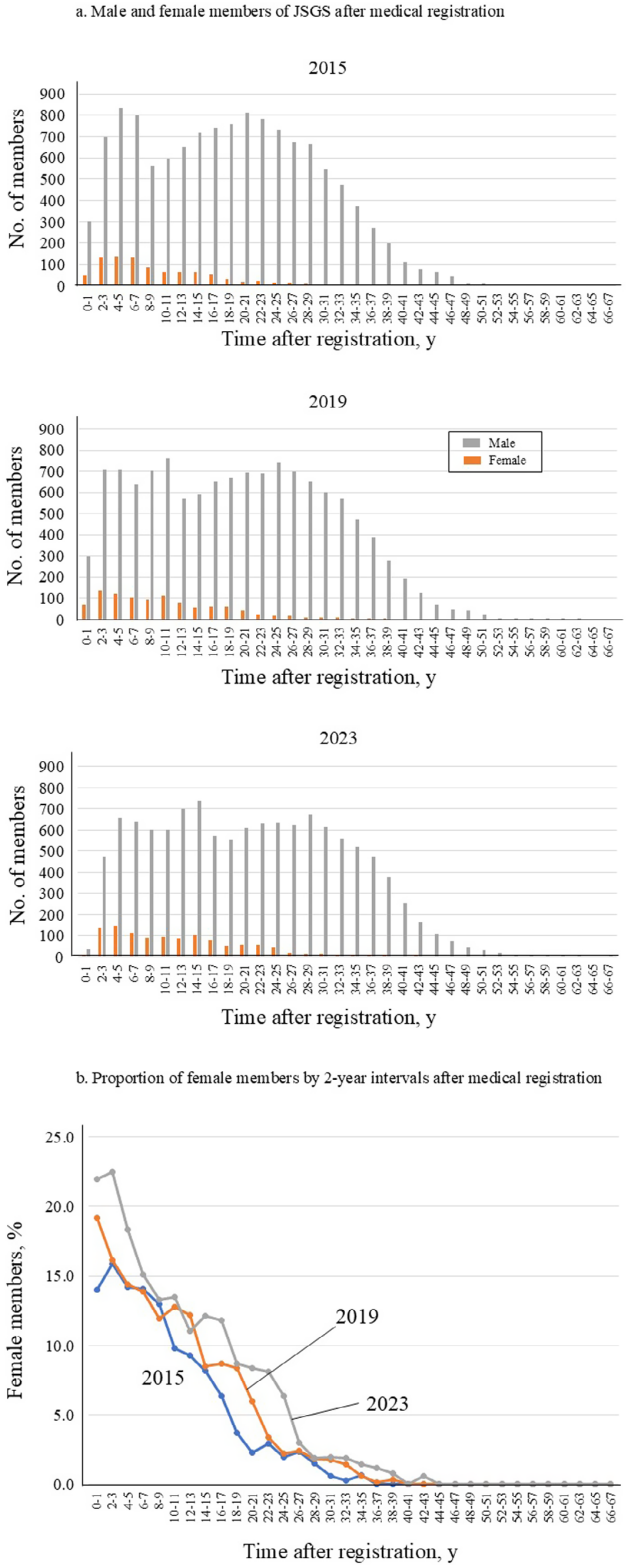
Male and female members of the Japanese Society of Gastrointestinal Surgery (JSGS) after medical registration (a); proportion of female JSGS members by 2‐year intervals after medical registration (b).

The total number of JSGS members in 2015 was 13 435, including 901 (6.7%) female and 12 534 (93.3%) male surgeons; in 2023, the total number of JSGS members was 13 139, with 1140 (8.7%) female and 11 999 (91.3%) male surgeons (Tables 1a and 1c). In 2023, the highest number of male members of JSGS occurs at 14–15 years after medical registration, while the number of female surgeons is the highest in less than 5 years of experience (Figure 1a). The highest percentage of female surgeons (22.5%) was found at 2–3 years of experience in 2023 (Figure 1b).

**TABLE 1a:** Operations performed by male and female surgeons in Japan in 2015.

	Total	Female, *n* (%)	Male, *n* (%)
Surgeons registered in the Japanese Society of Gastroenterological Surgery	13 435	901 (6.7)	12 534 (93.3)
Number of surgery performed			
All procedures	241 369	17 525 (7.3)	223 844 (92.7)
Appendectomy	45 914	4491 (9.8)	41 423 (90.2)
Cholecystectomy	109 705	8429 (7.7)	101 276 (92.3)
Right hemicolectomy	20 711	1375 (6.6)	19 336 (93.4)
Distal gastrectomy	34 060	1932 (5.7)	32 128 (94.3)
Low anterior resection	20 984	1024 (4.9)	19 960 (95.1)
Pancreaticoduodenectomy	9995	274 (2.7)	9721 (97.3)

### 3.2 Number and Percentage of Operations by Male and Female Surgeons

The proportion of operations performed by female surgeons was 7.3% in 2015 and 8.9% in 2019 (Tables 1a and 1b). Furthermore, 24 196 of 226 552 total operations (10.7%) were performed by female surgeons in 2023, which was increased in comparison with 2015 and 2019 (Table 1c). Among the six operative procedures investigated herein, the most frequently performed in 2023 by both male and female gastroenterological surgeons was cholecystectomy (111 190 total operations), followed by appendectomy (41 684 operations) and distal gastrectomy (24 459 operations). The proportion of operations performed by female surgeons was 13.6% (*n* = 5660) for appendectomy, 11.5% (*n* = 12 750) for cholecystectomy, 10.1% (*n* = 1920) for right hemicolectomy, 8.5% (*n* = 2082) for distal gastrectomy, 6.6% (*n* = 1259) for low anterior resection, and 4.7% (*n* = 525) for pancreaticoduodenectomy (Table 1c). Thus, female surgeons performed fewer high‐difficulty surgeries than their male counterparts.

**TABLE 1b:** Operations performed by male and female surgeons in Japan in 2019.

	Total	Female, *n* (%)	Male, *n* (%)
Surgeons registered in the Japanese Society of Gastroenterological Surgery	13 622	1031 (7.6)	12 591 (92.4)
Number of surgery performed			
All procedures	246 349	21 814 (8.9)	224 535 (91.1)
Appendectomy	46 856	5320 (11.4)	41 536 (88.6)
Cholecystectomy	117 663	11 327 (9.6)	106 336 (90.4)
Right hemicolectomy	20 445	1795 (8.8)	18 650 (91.2)
Distal gastrectomy	30 236	1944 (6.4)	28 292 (93.6)
Low anterior resection	19 883	1071 (5.4)	18 812 (94.6)
Pancreaticoduodenectomy	11 266	357 (3.2)	10 909 (96.8)

**TABLE 1c:** Operations performed by male and female surgeons in Japan in 2023.

	Total	Female, *n* (%)	Male, *n* (%)
Surgeons registered in the Japanese Society of Gastroenterological Surgery	13 139	1140 (8.7)	11 999 (91.3)
Number of surgery performed			
All procedures	226 552	24 196 (10.7)	202 356 (89.3)
Appendectomy	41 684	5660 (13.6)	36 024 (86.4)
Cholecystectomy	111 190	12 750 (11.5)	98 440 (88.5)
Right hemicolectomy	19 000	1920 (10.1)	17 080 (89.9)
Distal gastrectomy	24 459	2082 (8.5)	22 377 (91.5)
Low anterior resection	18 939	1259 (6.6)	17 680 (93.4)
Pancreaticoduodenectomy	11 280	525 (4.7)	10 755 (95.3)

**FIGURE 2:**
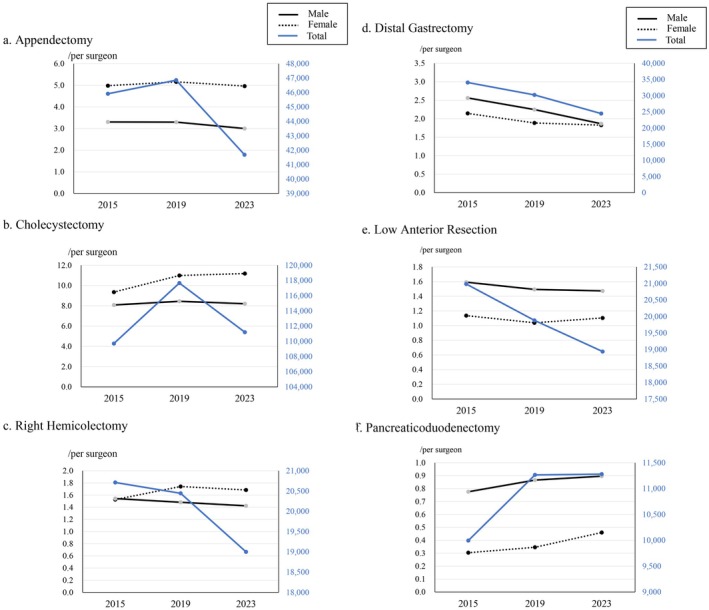
Chronological changes in the total number of operations and number of operations per male and female surgeons for six procedures: Appendectomy (a), cholecystectomy (b), right hemicolectomy (c), distal gastrectomy (d), low anterior resection (e), and pancreaticoduodenectomy (f).

Chronological changes in the total number of operations and number of operations per male and female surgeons for the above six procedures are shown in Figure 2a–f. The number of appendectomies and cholecystectomies performed per female surgeons was originally higher than that performed by male surgeons, and this trend remained unchanged from 2015 through 2023. The number of distal gastrectomies performed per male and female surgeons decreased, possibly owing to a reduction of the total number of distal gastrectomies; however, the reduction in the number of these surgeries per female surgeon was slightly smaller than the reduction among male surgeons. The number of pancreaticoduodenectomies performed by male and female surgeons increased over the study period with the increase in the total number of surgeries; however, high disparity remained between male and female surgeons.

### 3.3 Number of Operations According to Gender and Years of Experience

**FIGURE 3:**
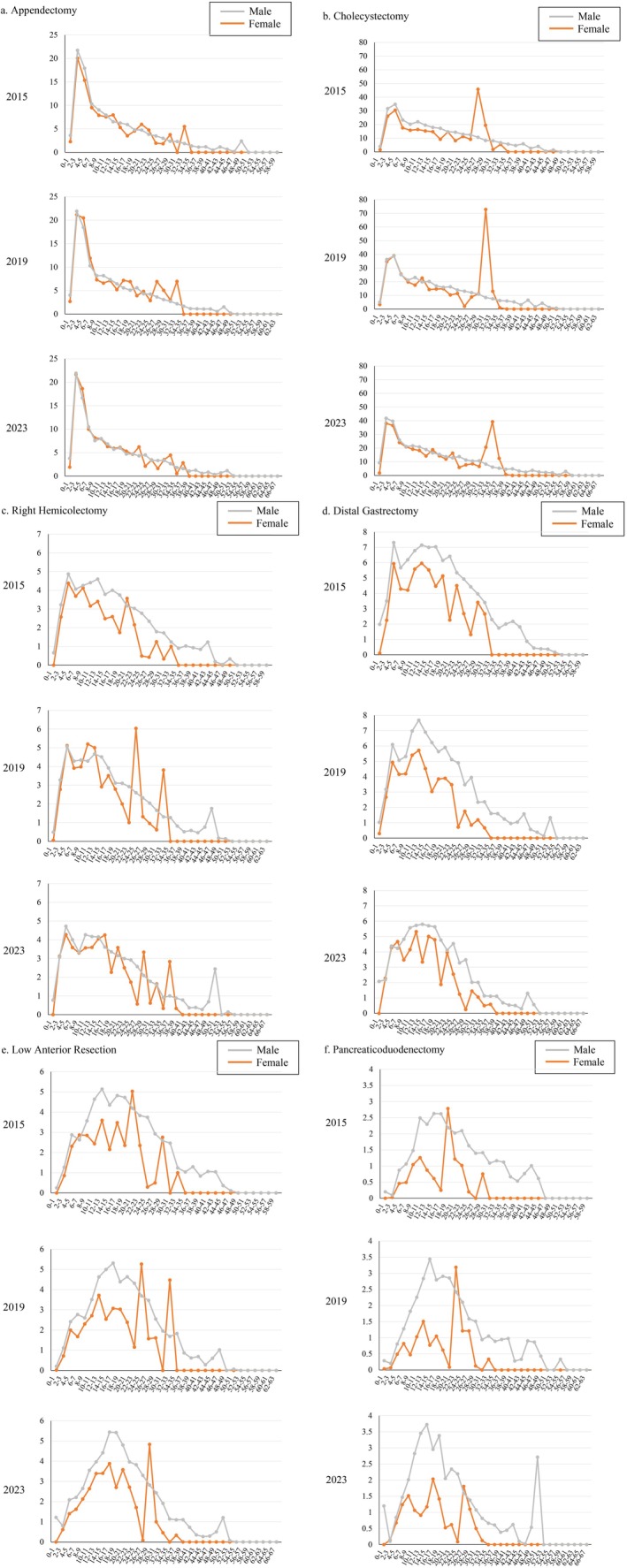
Number of operations per surgeon according to gender and every 2 years of experience for appendectomy (a), cholecystectomy (b), right hemicolectomy (c), distal gastrectomy (d), low anterior resection (e), and pancreaticoduodenectomy (f).

The number of operations in 2023, categorized according to surgeons' gender and 2‐year intervals since medical registration, is shown in Table S1. The number of operations per surgeon, based on every 2 years of surgical experience for each gender, is shown in Figure 3. The ratio of the number of operations per female and male surgeons is shown in Table 2.

**TABLE 2:** The difference between male and female in the number of operations per Surgeon.

Procedure	The ratio of female to male in the number of operations per surgeon																				
	0–1 years	2–3 years	4–5 years	6–7 years	8–9 years	10–11 years	12–13 years	14–15 years	16–17 years	18–19 years	20–21 years	22–23 years	24–25 years	26–27 years	28–29 years	30–31 years	32–33 years	34–35 years	36–37 years	38–39 years	≥ 40 years
Appendectomy																					
2015	0.635	0.923	0.857	0.924	0.875	0.953	1.222	0.846	0.594	0.949	1.253	1.237	0.559	0.609	1.600	0.000	2.921	0.000	0.000	0.000	0.000
2019	0.670	0.965	1.108	1.156	0.892	0.806	0.969	0.807	1.285	1.355	0.701	1.124	0.680	1.904	1.640	1.105	3.158	0.000	0.000	0.000	0.000
2023	0.505	0.988	1.118	0.954	1.078	0.994	0.920	1.035	1.024	1.128	0.984	1.442	0.472	1.022	0.494	1.049	1.695	0.291	1.778	0.000	0.000
Cholecystectomy																					
2015	0.390	0.826	0.876	0.751	0.784	0.740	0.784	0.825	0.534	0.994	0.569	0.878	0.733	4.297	2.329	0.206	0.818	0.000	0.000	0.000	0.000
2019	0.664	0.956	1.005	1.017	0.936	0.758	1.141	0.709	0.864	0.936	0.634	0.820	0.155	0.736	1.029	8.614	1.733	0.161	0.000	0.000	0.000
2023	0.192	0.911	0.923	0.931	0.988	0.887	0.874	0.753	1.139	0.936	0.870	1.266	0.427	0.686	0.812	0.617	2.502	6.433	2.359	0.147	0.000
Right hemicolectomy																					
2015	0.000	0.799	0.900	0.906	0.968	0.718	0.740	0.657	0.648	0.465	1.18	0.712	0.177	0.181	0.700	0.193	0.806	0.000	0.000	0.000	0.000
2019	0.109	0.846	1.009	0.912	0.917	1.212	1.072	0.646	0.894	0.895	0.643	0.342	2.323	0.567	0.469	0.373	2.907	0.000	0.000	0.000	0.000
2023	0.000	1.018	0.905	0.895	0.990	0.835	0.861	0.974	1.179	0.674	1.136	0.835	0.593	0.221	1.594	0.347	1.031	0.365	2.831	0.378	0.000
Distal gastrectomy																					
2015	0.054	0.643	0.812	0.757	0.680	0.822	0.834	0.789	0.636	0.836	0.354	0.842	0.543	0.297	0.862	0.783	0.000	0.000	0.000	0.000	0.000
2019	0.288	0.840	0.810	0.820	0.788	0.774	0.744	0.657	0.487	0.684	0.660	0.680	0.146	0.501	0.214	0.507	0.282	0.000	0.000	0.000	0.000
2023	0.000	1.044	0.975	1.104	0.721	0.744	0.929	0.576	0.879	0.851	0.395	0.968	0.561	0.378	0.072	0.718	0.526	0.439	0.523	0.000	0.000
Low anterior resection																					
2015	0.000	0.673	0.803	1.093	0.798	0.524	0.699	0.494	0.721	0.497	1.200	0.613	0.078	0.171	1.068	0.000	0.807	0.000	0.000	0.000	0.000
2019	0.094	0.646	0.828	0.606	0.885	0.773	0.803	0.508	0.578	0.690	0.514	0.268	1.426	0.454	0.632	0.000	2.660	0.000	0.000	0.000	0.000
2023	0.000	0.763	0.671	0.735	0.802	0.743	0.855	0.770	0.714	0.498	0.745	0.684	0.446	0.025	1.716	0.411	0.240	0.000	0.302	0.000	0.000
Pancreaticoduodenectomy																					
2015	0.000	0.279	0.528	0.461	0.705	0.506	0.381	0.233	0.095	1.271	0.602	0.486	0.122	0.000	0.533	0.000	0.000	0.000	0.000	0.000	0.000
2019	0.124	0.340	0.607	0.642	0.259	0.456	0.532	0.223	0.375	0.213	0.031	1.316	0.577	0.762	0.083	0.000	0.317	0.000	0.000	0.000	0.000
2023	0.000	1.317	0.800	0.846	0.754	0.377	0.263	0.315	0.689	0.419	0.255	0.265	0.041	1.129	0.796	0.465	0.153	0.000	0.000	0.000	0.000

#### 3.3.1 Low‐Difficulty Surgery: Appendectomy and Cholecystectomy

In 2023, the highest number of low‐difficulty operations performed by male surgeons was at 4–5 years after their medical registration; for female surgeons, the highest number of low‐difficulty surgeries performed was at 2–3 years after medical registration (Table S1). In 2015, 2019, and 2023, there were no gender differences in the number of appendectomies per surgeon in all groups according to years of experience (Figure 3a). The number of years of experience at which the number of appendectomies dropped to zero was higher in male surgeons than in female surgeons. In all study years, male surgeons performed appendectomies more than 50 years after their medical registration. In contrast, female surgeons did not perform appendectomies more than 34–35 years of experience in 2015 and 2019; this increased to 36–37 years of experience in 2023.

For cholecystectomy, female surgeons performed the most surgeries for protrusions at 28–29 years after medical registration in 2015, at 32–33 years in 2019, and at 36–37 years of experience in 2023. Male surgeons performed more cholecystectomies than female surgeons. However, the gender disparity younger than this female protrusion gradually decreased in 2019 and 2023 (Figure 3b).

#### 3.3.2 Medium‐Difficulty Surgery: Right Hemicolectomy and Distal Gastrectomy

The most right hemicolectomies were performed by male and female surgeons 4–5 years after medical registration (Table S1). In 2015, more male surgeons than female surgeons performed right hemicolectomies, except for in the group at 20–21 years of experience. This trend was more pronounced in the group at over 14 years of experience. The largest gender disparity was observed at 24–25 years of experience (female:male 0.177). In 2019, the number of operations performed by male and female surgeons appeared similar up to 18–19 years of experience (Figure 3c). The female: male ratio ranged from 0.8 to 1.3 between 2 and 19 years after medical registration, except for the group at 14–15 years of experience. In 2023, the number of right hemicolectomies per surgeon was similar up to 22–23 years of experience (Figure 3c). The female:male ratio ranged from 0.8 to 1.2 between 2 and 23 years of experience, except for in the group 18–19 years of experience (Table 2). Thus, the gender disparity for right hemicolectomy decreased yearly.

In 2015, the group performing the most distal gastrectomies was 4–5 years of experience for both male and female surgeons. In 2023, although the most distal gastrectomies performed by female surgeons were in this group, male surgeons performing the most distal gastrectomies had shifted to the group at 14–15 years of experience (Table S1). The gender disparity was more pronounced for distal gastrectomy compared with that for right hemicolectomy. In 2015 and 2019, the number of operations per male surgeons was greater than that by their female counterparts in all groups according to years of experience. In a comparison of operations performed by surgeons 10–19 years of experience, the gender disparity was larger in 2019 compared with 2015, but this was improved in 2023. In 2023, there was no gender disparity up to 7 years after medical registration (Figure 3d). The female:male ratio ranged from 0.9 to 1.2 for surgeons between 2 and 7 years of experience in 2023 (Table 2).

#### 3.3.3 High‐Difficulty Surgery: Low Anterior Resection and Pancreaticoduodenectomy

In 2015, female surgeons performed fewer low anterior resections compared with male surgeons at 8 or more years after medical registration, except for certain age groups in which the number of operations performed by some female surgeons was exceptionally high. In 2019 and 2023, more operations were performed by male surgeons than by female surgeons, except for protrusions (Figure 3e).

The number of pancreaticoduodenectomies performed by female surgeons was clearly lower than the number conducted by male surgeons in nearly all groups. In 2023, the gender disparity was less noticeable in the group up to 7 years of experience (Figure 3f). The female:male ratio for the number of operations per surgeon at 2–7 years of experience was between 0.8 and 1.3 in 2023 (Table 2). In 2015, male surgeons performed pancreaticoduodenectomies up to 44–45 years of experience whereas female surgeons did not perform pancreaticoduodenectomies beyond 30 years of experience. In 2023, male surgeons performed pancreaticoduodenectomies more than 50 years of experience, but female surgeons did not perform these surgeries more than 34 years of experience (Figure 3f). Thus, the difference between male and female surgeons for high‐difficulty surgeries was more pronounced than that for low‐ and medium‐difficulty surgeries.

## 4 Discussion

In this study, we examined the changes in gender disparity regarding surgeries performed among gastrointestinal surgeons in Japan before and after the implementation of measures to address gender inequality. Initiatives aimed at improving working environments for female surgeons were found to be effective, leading to an improving trend in the gender gap. However, gender disparities for high‐difficulty surgeries persisted.

Although the number of male JSGS members has been decreasing, the number of female members has been increasing [7]. The proportion of female gastrointestinal surgeons is over 20.0% among JSGS members younger than 30 years old. The JSGS has made efforts to improve the working environment for female surgeons under the president's leadership. The Gender Equality Working Group was established within the JSGS in September 2021 [8]. A new quota for female council members, proportional to the percentage of female JSGS members, was established in conjunction with the full council re‐election in 2025 [9]. The JSGS has promoted the participation of women on its board of directors and in positions of decision‐making and authority, as well as various committees. Additionally, the JSGS conducted a survey aimed at improving the working environment of surgeons to enable them to continue providing surgical services, even while raising children. An educational program to eliminate gender discrimination in surgical training has also been added.

A previous study demonstrated that female surgeons conducted fewer of the six types of operations assessed in our study, for all groups according to years after medical registration; this was particularly true for medium‐ and high‐difficulty surgeries [5]. The Hakodate Declaration states that the JSGS will support gender equality in surgical practice and will regularly review the number of operations performed by female and male gastrointestinal surgeons to eliminate gender disparities for medium‐difficulty surgeries. Additionally, the JSGS will continue to aim for providing equal opportunities in performing high‐difficulty surgeries [10]. In this study, we observed large improvements over the study period in the number of low‐difficulty surgeries performed per female surgeons, in all groups according to years of experience. Compared with other procedures, the protrusion observed in cholecystectomy was particularly prominent. One possible explanation for this protrusion may be that cholecystectomy, being a relatively common and low‐difficulty procedure, is consistently assigned to female surgeons even as they advance in their careers. Another possibility is that the surgeons who specialize to the laparoscopic cholecystectomy and work in the high‐volume centers for the laparoscopic surgeries are female. The number of medium‐difficulty surgeries performed by female surgeons showed an improving trend over the study period, yet some disparities remained. We identified large disparities remaining with respect to high‐difficulty operations. Recent reports from other countries such as the United States and New Zealand have also highlighted gender disparities in surgical case volume and autonomy [11, 12]. These findings suggest that such disparities are not unique to Japan but represent a broader international issue. Operator roles in high‐difficulty operations can be held among men long after surgical training. Pregnancy, childbirth, and childcare have an impact on the willingness to perform surgeries among female surgeons in these life stages [13, 14], who may not wish to conduct highly difficult operations. The burden from pregnancy through child‐rearing decreases over time, which could reduce the gender gap in surgeries performed; however, this reduction was not observed for high‐difficulty operations. It is important that female surgeons in the above life stages have the opportunity to conduct high‐difficulty surgeries, even partially, and to continue training. Another possibility is that high‐difficulty surgeries involve long working hours and many emergent treatments after surgery. For this reason, some female residents may not choose a subspeciality in gastrointestinal surgery [15, 16]. We also cannot rule out that in Japan, the assignment of a young surgeon's workplace is determined by their university medical office, which may have an influence on their experience conducting highly difficult surgeries [17]. Further improvements in gender disparity with regard to high‐difficulty surgical procedures are therefore needed.

In a comparison of distal gastrectomy performed by surgeons 10 and 19 years of experience, the gender disparity was larger in 2019 compared with 2015. In Japan, the technical safety of laparoscopy‐assisted distal gastrectomy for locally advanced gastric cancer has been demonstrated [18]. Consequently, the prevalence of laparoscopic gastrectomy for advanced gastric cancer has been increasing since 2015 [19, 20, 21]. Robot‐assisted gastrectomy, which had previously been performed as a self‐financed treatment or under advanced medical care, was covered by Japan's national health insurance in 2018 [22]. The increasing gender disparity for distal gastrectomy during 2015–2019 is likely attributable to the expansion of laparoscopic surgery and the adoption of robotic surgery. Similarly, robotic surgery for rectal cancer was introduced under the national insurance scheme at the same time as for gastric cancer. In our analysis, the gender disparity in rectal cancer surgeries per surgeon in 2019 had improved compared to 2015 among surgeons with less than 15 years of experience, but worsened in those with 16 or more years of experience. This trend is similar to what was observed in gastric cancer surgery. In a study on gender disparity in robotic surgical practice in a colorectal surgery training program, female residents had lower rates of console participation and fewer opportunities to complete total mesorectal excision than male residents [11]. However, as this interpretation remains hypothetical, further investigation is warranted to determine whether a gender bias exists in the performance of laparoscopic or robot‐assisted surgery.

Several efforts of the JSGS, including the Hakodate Declaration, have proven effective in leading to an improving trend in the gender gap in gastrointestinal surgery. However, gender disparities for high‐difficulty surgeries persist. It is hoped that further action will be taken with a view to the future of gastrointestinal surgery. First, it is important to create a flexible and efficient program by holding discussions with and addressing the needs of female surgeons who are raising children [23, 24, 25]. Furthermore, it is important to motivate the administrators of medical colleges and heads of surgical departments in all hospitals to eliminate discrimination in surgical training and place surgeons in training facilities without gender bias. Finally, it is necessary for the society as a whole to understand and address these situations.

The limitations of the present study are as follows. First, surgeons who had not participated in surgeries for a certain period was excluded in this study. However, those who participated in at least one procedure as either the primary surgeon or an assistant were included, which may not fully reflect the actual surgical practices of gastrointestinal surgeons in Japan. Second, data on members' time spent studying abroad, pregnancy, and childbirth are not recorded in the NCD or JSGS databases, potentially limiting the comprehensiveness of the analyses. Third, some JSGS members were not necessarily pursuing a career in gastrointestinal surgery, which may have influenced their surgeries performed. Fourth, the dataset did not contain information on the board certification status of individual surgeons in gastroenterological surgery. As board certification may influence the allocation of higher‐difficulty procedures, its absence may have affected our findings. Fifth, information regarding institutional accreditation status (e.g., by the Japanese Society of Hepato‐Biliary‐Pancreatic Surgery) and surgeons' subspecialty focus (e.g., upper GI, colorectal, or hepatobiliary‐pancreatic) was not available. These factors likely influence surgical case distribution and may have contributed to observed gender disparities.

## 5 Conclusions

As a result of various initiatives commenced by the JSGS in 2021, gender disparities were either eliminated or improved for low‐ and medium‐difficulty gastrointestinal surgeries. However, gender disparities persisted for high‐difficulty surgeries, indicating that continued efforts are needed to improve gender equality among gastrointestinal surgeons. Even in halfway, this is the first report that the society's efforts have corrected the gender gap.

## Author Contributions

**Chie Tanaka:** visualization, data curation, writing – original draft, writing – review and editing. **Hiroyuki Yamamoto:** supervision, formal analysis, data curation. **Sachiyo Nomura:** supervision, funding acquisition, writing – review and editing, project administration. **Emiko Kono:** funding acquisition, writing – review and editing. **Hideki Ueno:** supervision. **Yoshihiro Kakeji:** supervision. **Ken Shirabe:** supervision.

## Ethics Statement

All procedures were in accordance with the ethical standards of the responsible committee on human experimentation and in compliance with the Helsinki Declaration of 1964 and later versions. The internal review boards of participating institutions reviewed the scientific and ethical validity of the protocol.

## Conflicts of Interest

Hiroyuki Yamamoto is affiliated with the Department of Healthcare Quality Assessment at the University of Tokyo. The department is a social collaboration department supported by grants from the National Clinical Database, Intuitive Surgical Sarl, Johnson & Johnson K.K., and Nipro Co. And Dr. Chie Tanaka, Dr. Sachiyo Nomura, Dr. Hideki Ueno, Dr. Yoshihiro Kakeji and Dr. Ken Shirabe are current editorial members.

## Supporting information


**Table S1:** Number of Operations Categorized by Surgeons' Gender and Every 2 Years after Medical Registration in 2015.

## Data Availability

Research data are not shared. Data from the National Clinical Database (NCD) in Japan was used in this study.
