# Supplementary material for: Trends in Gender Disparities in Surgical Experience: A National Clinical Database Study
Source: Ann Gastroenterol Surg. 2025 Aug 23;10(1):263–74. doi: 10.1002/ags3.70080 (PMC12757170; doi:10.1002/ags3.70080)
Supplement: Supplementary file 1 — Table S1: Number of Operations Categorized by Surgeons' Gender and Every 2 Years after Medical Registration in 2015. [file AGS3-10-263-s001.docx]

| Procedure | Number of operations | | | | | | | | | | | | | | | | | | | | |
| --- | --- | --- | --- | --- | --- | --- | --- | --- | --- | --- | --- | --- | --- | --- | --- | --- | --- | --- | --- | --- | --- |
|  | 0-1 y | 2-3 y | 4-5 y | 6-7 y | 8-9 y | 10-11 y | 12-13 y | 14-15 y | 16-17 y | 18-19 y | 20-21 y | 22-23 y | 24-25 y | 26-27 y | 28-29 y | 30-31 y | 32-3 y | 34-35 y | 36-37 y | 38-39 y | ≥40 y |
| Appendectomy |  | | | | | | | | | | | | | | | | | | | | |
| Female | 73 | 1385 | 1063 | 621 | 331 | 245 | 260 | 175 | 89 | 66 | 57 | 64 | 20 | 15 | 21 | 0 | 6 | 0 | 0 | 0 | 0 |
| Male | 432 | 7839 | 7515 | 4115 | 2550 | 2380 | 2123 | 2254 | 2184 | 1814 | 1938 | 1506 | 1268 | 1010 | 783 | 634 | 447 | 253 | 161 | 117 | 100 |
| Cholecystectomy |  | | | | | | | | | | | | | | | | | | | | |
| Female | 55 | 1820 | 2105 | 1136 | 668 | 532 | 512 | 484 | 233 | 209 | 89 | 147 | 66 | 303 | 59 | 5 | 6 | 0 | 0 | 0 | 0 |
| Male | 433 | 11518 | 14603 | 9317 | 5711 | 6615 | 6362 | 6444 | 6361 | 5562 | 5860 | 5070 | 4571 | 3573 | 2786 | 2221 | 1598 | 1072 | 654 | 600 | 345 |
| Right hemicolectomy |  | | | | | | | | | | | | | | | | | | | | |
| Female | 0 | 184 | 304 | 241 | 173 | 103 | 115 | 82 | 65 | 25 | 41 | 27 | 5 | 4 | 4 | 1 | 1 | 0 | 0 | 0 | 0 |
| Male | 45 | 1197 | 2036 | 1623 | 1204 | 1320 | 1496 | 1364 | 1481 | 1419 | 1286 | 1186 | 1009 | 790 | 597 | 473 | 292 | 170 | 141 | 94 | 113 |
| Distal gastrectomy |  | | | | | | | | | | | | | | | | | | | | |
| Female | 4 | 161 | 413 | 282 | 176 | 181 | 206 | 183 | 112 | 72 | 20 | 64 | 23 | 9 | 18 | 8 | 0 | 0 | 0 | 0 | 0 |
| Male | 119 | 1305 | 3059 | 2266 | 1749 | 2037 | 2325 | 2526 | 2609 | 2330 | 2611 | 2098 | 1795 | 1488 | 1327 | 935 | 554 | 327 | 285 | 211 | 172 |
| Low anterior resection |  | | | | | | | | | | | | | | | | | | | | |
| Female | 0 | 61 | 160 | 188 | 120 | 80 | 121 | 70 | 88 | 33 | 53 | 32 | 3 | 4 | 10 | 0 | 1 | 0 | 0 | 0 | 0 |
| Male | 29 | 471 | 1204 | 1048 | 1010 | 1391 | 1672 | 1571 | 1788 | 1790 | 1704 | 1511 | 1359 | 986 | 860 | 675 | 304 | 196 | 182 | 84 | 125 |
| Pancreaticoduodenectomy |  | | | | | | | | | | | | | | | | | | | | |
| Female | 0 | 2 | 32 | 31 | 47 | 41 | 31 | 22 | 6 | 34 | 10 | 13 | 2 | 0 | 3 | 0 | 0 | 0 | 0 | 0 | 0 |
| Male | 5 | 36 | 363 | 423 | 417 | 747 | 748 | 948 | 970 | 829 | 825 | 829 | 598 | 472 | 470 | 300 | 265 | 212 | 95 | 55 | 114 |

Table S1. Number of Operations Categorized by Surgeons' Gender and Every 2 Years After Medical Registration in 2015

| Procedure | Number of operations | | | | | | | | | | | | | | | | | | | | |
| --- | --- | --- | --- | --- | --- | --- | --- | --- | --- | --- | --- | --- | --- | --- | --- | --- | --- | --- | --- | --- | --- |
|  | 0-1 y | 2-3 y | 4-5 y | 6-7 y | 8-9 y | 10-11 y | 12-13 y | 14-15 y | 16-17 y | 18-19 y | 20-21 y | 22-23 y | 24-25 y | 26-27 y | 28-29 y | 30-31 y | 32-33 y | 34-35 y | 36-37 y | 38-39 y | ≥40 y |
| Appendectomy |  | | | | | | | | | | | | | | | | | | | | |
| Female | 136 | 1491 | 1228 | 613 | 336 | 372 | 283 | 145 | 212 | 209 | 88 | 61 | 24 | 55 | 28 | 16 | 23 | 0 | 0 | 0 | 0 |
| Male | 528 | 7928 | 6523 | 3278 | 2882 | 3114 | 2106 | 1916 | 1818 | 1711 | 1930 | 1490 | 1565 | 1280 | 997 | 820 | 632 | 401 | 222 | 155 | 240 |
| Cholecystectomy |  | | | | | | | | | | | | | | | | | | | | |
| Female | 171 | 2453 | 2349 | 1316 | 960 | 983 | 901 | 389 | 447 | 454 | 231 | 136 | 17 | 84 | 59 | 328 | 46 | 3 | 0 | 0 | 0 |
| Male | 650 | 13140 | 13723 | 8023 | 7467 | 8800 | 5700 | 5984 | 5509 | 5324 | 5634 | 4791 | 4846 | 4227 | 3541 | 2551 | 2151 | 1469 | 1119 | 735 | 952 |
| Right hemicolectomy |  | | | | | | | | | | | | | | | | | | | | |
| Female | 3 | 197 | 309 | 199 | 183 | 292 | 199 | 79 | 107 | 80 | 44 | 12 | 54 | 15 | 7 | 4 | 11 | 0 | 0 | 0 | 0 |
| Male | 56 | 1191 | 1789 | 1363 | 1530 | 1630 | 1337 | 1338 | 1277 | 1043 | 1078 | 1010 | 959 | 814 | 665 | 499 | 376 | 299 | 155 | 72 | 169 |
| Distal gastrectomy |  | | | | | | | | | | | | | | | | | | | | |
| Female | 13 | 190 | 291 | 213 | 201 | 305 | 228 | 122 | 100 | 117 | 84 | 40 | 6 | 18 | 5 | 7 | 4 | 0 | 0 | 0 | 0 |
| Male | 93 | 1151 | 2152 | 1618 | 1872 | 2651 | 2201 | 2045 | 2025 | 1888 | 2032 | 1765 | 1807 | 1218 | 1281 | 706 | 680 | 383 | 308 | 172 | 244 |
| Low anterior resection |  | | | | | | | | | | | | | | | | | | | | |
| Female | 1 | 51 | 117 | 86 | 111 | 152 | 147 | 69 | 92 | 92 | 54 | 14 | 49 | 14 | 11 | 0 | 11 | 0 | 0 | 0 | 0 |
| Male | 21 | 404 | 851 | 881 | 916 | 1337 | 1326 | 1479 | 1729 | 1470 | 1612 | 1488 | 1362 | 1221 | 822 | 589 | 482 | 426 | 169 | 87 | 140 |
| Pancreaticoduodenectomy |  | | | | | | | | | | | | | | | | | | | | |
| Female | 1 | 5 | 29 | 42 | 26 | 57 | 60 | 21 | 35 | 20 | 2 | 36 | 9 | 11 | 1 | 0 | 2 | 0 | 0 | 0 | 0 |
| Male | 36 | 74 | 287 | 409 | 640 | 861 | 811 | 1020 | 908 | 973 | 986 | 835 | 781 | 565 | 490 | 285 | 301 | 211 | 177 | 136 | 123 |

Number of Operations Categorized by Surgeons' Gender and Years After Medical Registration in 2019

| Procedure | Number of operations | | | | | | | | | | | | | | | | | | | | |
| --- | --- | --- | --- | --- | --- | --- | --- | --- | --- | --- | --- | --- | --- | --- | --- | --- | --- | --- | --- | --- | --- |
|  | 0-1 y | 2-3 y | 4-5 y | 6-7 y | 8-9 y | 10-11 y | 12-13 y | 14-15 y | 16-17 y | 18-19 y | 20-21 y | 22-23 y | 24-25 y | 26-27 y | 28-29 y | 30-31 y | 32-33 y | 34-35 y | 36-37 y | 38-39 y | ≥40 y |
| Appendectomy |  | | | | | | | | | | | | | | | | | | | | |
| Female | 19 | 1548 | 1372 | 566 | 376 | 381 | 272 | 304 | 232 | 140 | 130 | 173 | 45 | 34 | 11 | 22 | 24 | 3 | 8 | 0 | 0 |
| Male | 77 | 5402 | 5482 | 3349 | 2279 | 2406 | 2391 | 2109 | 1701 | 1308 | 1445 | 1358 | 1426 | 1047 | 1113 | 1035 | 740 | 477 | 376 | 199 | 304 |
| Cholecystectomy |  | | | | | | | | | | | | | | | | | | | | |
| Female | 17 | 2798 | 2690 | 1371 | 966 | 911 | 795 | 729 | 714 | 377 | 354 | 451 | 128 | 72 | 48 | 39 | 82 | 169 | 37 | 2 | 0 |
| Male | 176 | 10481 | 13050 | 8279 | 6384 | 6531 | 7294 | 6977 | 4698 | 4277 | 4207 | 4043 | 4384 | 3530 | 3544 | 3317 | 2320 | 1593 | 1250 | 843 | 1262 |
| Right hemicolectomy |  | | | | | | | | | | | | | | | | | | | | |
| Female | 0 | 240 | 316 | 204 | 153 | 168 | 150 | 207 | 162 | 61 | 98 | 69 | 38 | 5 | 27 | 4 | 8 | 1 | 8 | 1 | 0 |
| Male | 11 | 803 | 1551 | 1277 | 1002 | 1288 | 1457 | 1534 | 1026 | 931 | 961 | 943 | 931 | 801 | 706 | 548 | 442 | 242 | 236 | 165 | 225 |
| Distal gastrectomy |  | | | | | | | | | | | | | | | | | | | | |
| Female | 0 | 176 | 317 | 264 | 161 | 194 | 233 | 171 | 190 | 125 | 56 | 110 | 55 | 12 | 1 | 8 | 5 | 2 | 2 | 0 | 0 |
| Male | 13 | 568 | 1441 | 1350 | 1447 | 1674 | 2000 | 2140 | 1627 | 1567 | 1454 | 1296 | 1427 | 1028 | 1170 | 625 | 567 | 296 | 267 | 208 | 212 |
| Low anterior resection |  | | | | | | | | | | | | | | | | | | | | |
| Female | 0 | 49 | 104 | 93 | 98 | 125 | 142 | 174 | 147 | 70 | 97 | 75 | 37 | 1 | 39 | 5 | 2 | 0 | 1 | 0 | 0 |
| Male | 5 | 210 | 684 | 703 | 798 | 1070 | 1385 | 1629 | 1546 | 1504 | 1464 | 1246 | 1219 | 1030 | 947 | 754 | 535 | 299 | 261 | 206 | 185 |
| Pancreaticoduodenectomy |  | | | | | | | | | | | | | | | | | | | | |
| Female | 0 | 11 | 49 | 71 | 70 | 49 | 42 | 58 | 77 | 38 | 15 | 18 | 2 | 15 | 5 | 4 | 1 | 0 | 0 | 0 | 0 |
| Male | 2 | 29 | 276 | 465 | 611 | 855 | 1207 | 1372 | 841 | 933 | 627 | 737 | 692 | 500 | 468 | 337 | 229 | 174 | 143 | 76 | 181 |

Number of Operations Categorized by Surgeons' Gender and Years After Medical Registration in 2023
